# Unearthing roundworms: Nematodes as determinants of human health

**DOI:** 10.1016/j.onehlt.2025.101103

**Published:** 2025-06-13

**Authors:** Lisa van Sluijs, Stefan Geisen, Jose L. Lozano-Torres, Mark G. Sterken, Mariëtte T.W. Vervoort, Ruud H.P. Wilbers

**Affiliations:** Laboratory of Nematology, Wageningen University & Research, Wageningen, the Netherlands

**Keywords:** Nematodes, Human health, Model organisms, Education

## Abstract

Nematodes are all around us, both in the environment and associated to all imaginable organisms, ranging from plants to animals and humans. As such, nematodes are intimately linked to human, animal and plant health, both directly and indirectly, positively and negatively. Historically, studies mainly focused on the direct negative impact nematodes induce. As a result, we lack a comprehensive overview on the importance of nematodes – including non-direct and positive links. Therefore, we holistically report how human health is affected by direct interactions with nematodes, and indirect interactions via plants, animals and the environment. For that, we first focus on nematodes as human pests, but also show that some of these human-associated nematodes can positively affect human immunity. Subsequently, we summarize how nematodes impair food production and the environment as animal and plant pests, followed by an overview of the multiple indirect links that often benefit human health by enhancing animal and plant performance. Finally, we highlight the different opportunities to improve human health with nematodes, emerging from nematodes as model organisms, as a source for immunomodulatory proteins and as biological products in agriculture. We believe that our review will foster studies on links between nematodes and human health that we believe are highly promising to advance science and technology.

## 1 Introduction

Human health is determined by a vast complexity of factors, including interactions with other organisms with beneficial or detrimental outcomes [1]. Having gone through the severe covid-19 pandemic, the acknowledgment of the importance of diseases for human health is imprinted in the world's population. Viruses (e.g. the common flu), bacteria (e.g. *Staphylococcus aureus*), fungi (e.g. *Aspergillus*), and protists (e.g. the malaria-causing *Plasmodium falciparum*) are notorious human pathogens. Nematodes are known agents of human disease, they are equally widespread and can cause major threats [2].

A diversity of nematodes directly infects and reduces human fitness [2] and historically most research on nematodes focused on these species with detrimental effects [3]. In addition, parasitic nematodes cause indirect damage to human health by infecting animals and plants. Animal – including human – parasitic nematodes usually have a narrow host range, while still virtually all animals can be infected by at least one parasitic nematode species [4]. In contrast, multiple species of plant-parasitic nematodes have a broad host range with at least hundreds of potential hosts throughout the plant kingdom, but also more specialized plant-parasitic nematodes exist [5].

Despite the wide relevance as direct and indirect threats to human health, there is more to nematodes. The vast majority of nematode species – being potentially in the millions [6,7] – are free-living. These aquatic and soil-dwelling animals do not cause disease, and many likely are beneficial to plant and animal health. Nematodes are by far the most abundant animals on Earth, with the famous biologist E.O. Wilson stating that ‘*four out of five animals on Earth are nematode worms – if all solid materials except nematode worms were to be eliminated, you could still see the ghostly outline of most of it in nematode worms*’. Only soil nematodes have 60 billion times more individuals than humans [8], with aquatic nematodes likely adding a similar number of individuals [9]. This environmental nematode diversity is linked to human health by its integration in soil food webs and its role in catalyzing nutrient cycling [10,11].

Nematodes also have a value for science and education, which links to knowledge-gains and human health. Because of their relatively simple biology, researchers have used nematodes to gain fundamental insights into cell and molecular biology, mainly using the model species *Caenorhabditis elegans* [12]. This research has provided the foundation for finding answers to improve human health by understanding and consequently fighting diseases such as cancer and neurodegenerative diseases [13]. Nematodes have valuable characteristics such as fast reproduction, microscopic transparency and behavioral responses, making nematodes convenient study models for researchers, and also students. Therefore, nematode models are broadly applied in education and many researchers in human health have learned to perform biological experiments using a nematode for the first time.

**Fig. 1:**
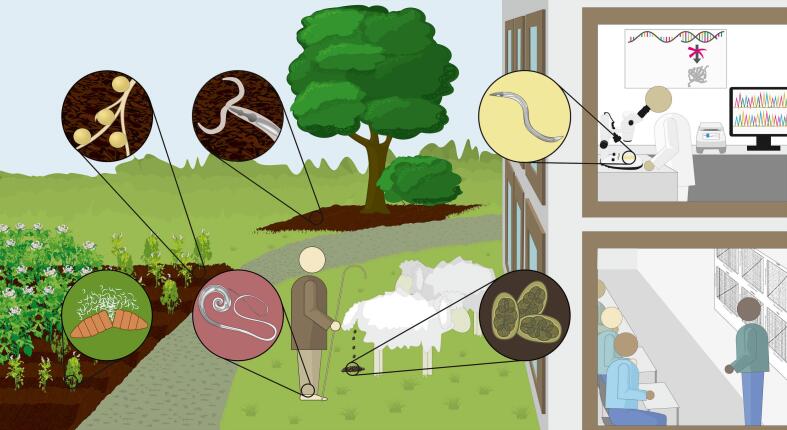
The direct and indirect effects of nematodes on human health. Clockwise description of the circles, starting on the top-left: 1) Plant-parasitic nematodes such as *Globodera* and *Meloidogyne* infect virtually all of the crops humans rely on for food. 2) In natural ecosystems, nematodes cover many trophic levels, from bacterivorous or fungivorous to predatory (as the depicted *Pristionchus*). 3) Nematodes, especially *Caenorhabditis elegans,* have been extensively used as research model and have driven many advances in the field of biology. Nematodes are also great for use in teaching, and can be used to illustrate some biological concepts for children at primary school level. 4) Animals can be infected by nematodes, and grazing ruminants (like sheep) especially suffer from gastrointestinal worms. 5) Humans can be infected by a diverse range of nematodes, including (the now almost eradicated) *Dracunculus medinensis*. 6) Nematodes can also parasitize invertebrates (like insects) and some of these are applied in biocontrol, like *Steinernema*.

Here, we comprehensively report how nematodes directly and indirectly affect human health (Fig. 1). As may be expected from such an extensively diverse phylum, the described effects of nematodes on human health often contain negative and positive aspects, and contrary to most, we integrate both aspects in this overview. The first section will focus on the impact of nematode species that parasitize humans and thus have a directly measurable effect on human health. The second section provides an overview of how nematodes indirectly affect human health by their impact on food production and the environment. The third section provides examples of how nematodes are applied to benefit human health, including their source for immunomodulatory proteins with therapeutic applications. Finally, we describe how nematodes offer different opportunities to improve human health, e.g. as model organisms in research and education.

## 2 Direct impact of nematodes on human health

Nematodes have a direct impact on human and animal health. In this section, we describe the impact that nematode infections can have on human health and the mechanisms involved in these infections. In addition to describing the malign effects, we illustrate how we are now starting to identify beneficial effects of nematode infections on our immune system.

### 2.1 Parasitic nematodes – a major burden on human health

Parasitic nematodes directly impact human health as they infect over 1.5 billion people worldwide [14]. Due to global warming and globalization, diverse parasites are expanding their geographic range to new regions posing more humans at risk for infection [15]. These parasites are mostly found in places where sanitation is poor. Infections with nematodes can occur via the oral-faecal route (whipworms, like *Trichuris* spp., and roundworms, like *Ascaris lumbricoides*), active penetration of the skin (hookworms, like *Ancylostoma* spp. and *Necator americanus*) or ingestion of undercooked meat (*Trichinella spiralis*) or fish (*Anisakis*). In addition, some nematode species rely on arthropods as intermediate hosts and invade humans after an insect bite (filarial nematodes, including *Onchocerca volvulus, Wuchereria bancrofti* and *Brugia spp*), or upon ingestion of water that is contaminated with infected copepods (guinea worm, *Dracunculus medinensis*). Parasitic nematodes rely on a live host to complete their lifecycle and therefore do not tend to kill their definitive host (overall fatality rate < 1 % [16,17]). Still, the combined impact of individual parasite species (reviewed in [18]) is as devastating as for other parasitic diseases, such as malaria [19].

Parasitic nematodes burden human health via several distinct mechanisms. Most nematode species that infect humans colonize and reproduce within the gut, where they deposit eggs in the feces. These infections often occur asymptomatically, but when nematodes are present in large numbers they can compete for nutritional resources or obstruct the gut [20]. This can ultimately lead to severe morbidity, including persistent diarrhea, anemia, abdominal swelling, and deficiencies in iron and vitamins. Furthermore, malnutrition in children can impair their growth and cognitive development [20,21]. In addition, some nematode species cause physical trauma during invasion (e.g. creeping eruptions caused by hookworms), or when migrating to other organs during specific life stages (e.g. blindness caused by migration to the eyes by *O. volvulus*). Other nematode infections can lead to abnormal growth of body parts, which is seen for certain filarial nematodes [14,22,23]. Such body deformities can affect the mental health of patients and could lead to social stigmatization, loss of employment and increased medical expenses.

Although periodic mass administration of anthelmintic drugs is usually well-suited to prevent or treat nematode infections, in some endemic countries including ones in Latin America, sub-Saharan Africa and Southeast Asia, more than half of the population still suffers from these parasites as reinfections easily occur [24]. In addition, the emergence of anthelmintic drug resistance in parasitic nematodes could be a major concern. Anthelmintic drug resistance in parasites of sheep, goats and cattle is already posing a significant threat to global livestock farming [25] and is emerging in companion animals [26]. So far, there seems to be little evidence to support the development of drug resistance in human parasites, but nucleotide polymorphisms associated with anthelmintic drug resistance in animal parasites are occurring at low frequency in human parasites [27]. For the detection and monitoring of drug resistance we rely on proper diagnostic tools, but commercial tests are still unavailable.

### 2.2 Parasitic nematodes – not only the bad and ugly

Besides the negative impact of nematode infections on human health, nematodes are slowly gaining a more positive image as being ‘old friends’. This idea originates from the observations that early-life exposure to common childhood infections (including nematodes) can mitigate the development of allergies and autoimmune diseases [28,29]. In industrialized countries the incidence of nematode (and other) infections has reduced tremendously by realizing proper sanitation, improved hygiene and treatment regimes. Simultaneously, the incidence of allergies and autoimmune diseases, like multiple sclerosis and type 1 diabetes, has increased [30] with approximately 5 % of the human population being affected by an autoimmune disease at some point in their life [31]. Co-evolution between host and parasite seems vital for the development of immunological regulatory networks that tolerate these ‘old friends’ and limit damage to host tissues due to excessive immune responses [29]. These regulatory networks prevent the immune system to respond to harmless environmental antigens or self-antigens, thereby protecting people from allergies or autoimmune diseases.

## 3 Indirect effects of nematodes on human health

Nematodes have an indirect impact on human health by infecting animals and plants. In this section, we focus on how animal- and plant-parasitic nematodes pose a risk as zoonotic infections (transmission from animals to humans) or threaten food production. Furthermore, we describe the key role of non-pathogenic nematodes in the global nutrient cycling and how environmental changes shifting nematode populations may affect human health.

### 3.1 Infections of animal-parasitic nematodes in livestock and companion animals

Infections with animal-parasitic nematodes (APNs) are common in animals and negatively affect their health, wellbeing, and productivity for livestock animals [32]. Common examples of APNs in livestock are gastrointestinal nematodes (*Ascaris suum*, *Ostertagia ostertagi, Cooperia oncophora, Teladorsagia circumcincta, Haemonchus contortus and Trichostrongylus*)*,* the bovine lungworm (*Dictyocaulus viviparus*), and *Trichinella spiralis,* the latter being a zoonotic parasite with a broad host-range. Zoonotic parasites are also found in fish (Anisakis) [33], as well as domestic animals (*Toxocara* spp. and *Ancylostoma caninum)* [34] and rodents (*Angiostrongylus cantonensis*) [35]. The prevalence of APN infections varies worldwide and is determined by environmental conditions (including climate), age of the animal, host susceptibility, and livestock management systems [36], or display of predation behaviour by companion animals [34]. APNs have are transmitted via the oral-faecal route. At high worm burden, the parasites can cause anemia, lethargy, diarrhea, but also affect feed consumption and food conversion of the animal, which directly impacts livestock productivity [36]. For grazing ruminants, the combined annual economic impact of common worm parasites (including gastrointestinal nematodes, lungworms and liver fluke (the trematode *Fasciola hepatica*)) has been estimated at €1.8 billion for 18 European countries, of which 81 % is caused by production losses and 19 % by treatment costs [36].

Infections with APNs are currently controlled by the periodic administration of anthelmintic drugs belonging to four major classes, but development of resistance against all four classes of drugs has been reported [37]. An additional concern for the extensive use of anthelmintics is that there is insufficient knowledge and public awareness about the negative (or toxic) effects of these drugs on the environment [37]. Anthelmintics are released into the environment via feces or urine by the infected animals and are distributed in open water and soil, where they can affect various organisms directly (e.g. plants, soil invertebrates and water animals) or indirectly via the food-chain. Future efforts should investigate the impact of anthelminthics (and derivatives thereof) in ecosystems and raise public awareness about the overuse and potential side-effects caused by anthelmintics.

An alternative and more durable strategy to combat APN would be through vaccination. However, to date only two commercially available vaccines against APN exist, which target bovine lungworm *Dictyocaulus viviparus* (Bovilis Huskvac™) or the sheep gastrointestinal nematode *Haemonchus contortrus* (Barbervax™/Wirevax™). Both vaccines rely on the deliberate infection of animals to obtain larvae or crude antigens for vaccine preparation, an approach that is unsustainable for large scale application. Therefore, the production of recombinant subunit vaccines has been evaluated against a range of gastrointestinal nematodes, but only very few induced sufficient levels of protection [38,39]. One of the major challenges is the choice of expression system. Where several native antigens isolated from the parasite show promise in inducing protection, the recombinant counterparts expressed in for instance *Escherichia coli* or *Pichia pastoris* fail to protect the animals [39]. A likely reason for this is that many established expression systems differ in their post-translational modifications, such as glycosylation, in comparison to the parasite. The importance of glycosylation in vaccine development has recently been shown in a proof-of-concept study, where the protective capacity of a recombinant antigen of *Ostertagia ostertagi* was restored by mimicking its native N-glycosylation in a plant-based expression system [40]. This new insight provides a useful framework for developing recombinant vaccines targeting other parasitic nematodes.

### 3.2 Agricultural losses caused by plant-parasitic nematodes

Plant-parasitic nematodes (PPNs) can severely damage plants and reduce crop yields [41]. These damages and losses caused by PPNs largely depend on the crops produced, the nematode species present and the initial nematode population density [42]. In extreme cases where susceptible crop plants are exposed to fields infested with virulent nematodes, and poor agricultural management practices, crop losses can surpass 80 % of the total production [43]. Global estimates of agricultural crop losses due to the direct damage induced by PPNs indicate a 15 % crop loss [41,44], which amounts to hundreds of billions of annual damages [37].

Besides the direct damage on food production caused by PPNs, some PPN species are disease vectors of plant-infecting viruses [45] or cause secondary plant infections by bacterial, fungal, and other pathogens [42,43]. Moreover, the increasing threat PPNs pose to human health and global food production is also due to methods used to manage plant-parasitic nematodes. Although the most harmful pesticides used to control nematodes face a global ban [46], nematicides are still applied in agricultural production. Nematicides can cause health problems in humans, contamination of soil and water, and have severe impacts on the environmental biodiversity [47,48]. The use of resistance genes is the most used alternative to pesticides for managing plant-parasitic nematodes in agricultural production [45,46]. Some of the most widely applied nematode resistance genes, e.g. Mi1 [[49], [50], [51]] stop working when the plants are grown in warmer conditions [52]. Current global warming conditions therefore undermines the most important strategy to combat parasitic nematodes in an environmentally friendly manner [[51], [52], [53], [54]].

### 3.3 Functional importance of environmental nematodes and their impact on humans

Human health is intimately tied to the environment as evidenced by increased stress and mortality rates in polluted environments [53,54]. Also, biotic components in soils and aquatic habitats influence environmental and human health [55,56]. Due to the immense taxonomic diversity of nematodes of potentially millions of species [57] that is mirrored in a wide array of environmental functions, nematodes also link to human health. By far most nematodes are non-pathogenic. These nematodes have evolved to prey on all kinds of organisms including, among others, bacteria, fungi and animals. Indeed, nematodes are placed in virtually all positions in food webs [11,58], resulting in a major importance for the global nutrient cycling. For example, nematodes increase plant growth by enhancing plant nutrient uptake [59,60], such as of phosphorous [61,62]. Nematodes also facilitate nitrogen uptake by plants through predating the microbiome [60]. Finally, nematodes have a profound role in carbon cycling via affecting the microbiome, with potential direct influence on climate change [[63], [64], [65]]. Indeed, carbon cycling is enhanced by nematodes [66] and greenhouse gas emissions positively correlate with increasing numbers of nematodes [67]. Taken together, without nematodes nutrient transfer from microbes up the food chain and to plants would be impeded [11]. Consequences for human health would include malnutrition and minimization of natural products due to reduced plant performance and animal biodiversity. Yet, we know comparatively little on the links of non-pathogenic nematodes with human health and how they are changing in the future. This knowledge gap warrants further research.

## 4 Nematodes in application

Many attributes of the phylum Nematoda, including its taxonomic and functional diversity, contribute to a variety of direct and indirect links to human health. Here we describe how these traits can offer opportunities to use nematodes as immunomodulators, biocontrol agents against plant pests, as bioindicators for soil and environmental health, and as models in ecotoxicology.

### 4.1 Harnessing the immunomodulatory potential of nematodes for treatment of human disease

The ‘old friends’ observation that the loss of parasite infections increases the risk for the development of inflammatory diseases has spiked parasitology research towards applications for worm therapy [68]. In 2005, a first clinical trial with live nematode infection was commenced to study the safety and efficacy of worm therapy using eggs of the porcine whipworm *Trichuris suis* that temporarily infect the human gut [69]. In this trial, a reduced severity of Crohn's disease was observed for 79 % of the patients [69]. These findings led to several follow-up clinical trials with *T. suis* ova as well as controlled infection with the human hookworm *Necator americanus* in patients with inflammatory bowel diseases, multiple sclerosis, rheumatoid arthritis, allergic rhinitis, asthma or celiac disease [70] often showing clinical improvements, yet efficacy of the therapies varied. Clinical improvements found after worm therapy rely on: 1) enhanced secretion of anti-inflammatory cytokines, 2) promotion of the development of regulatory T and B cells, 3) stimulation of alternative activation of macrophages, and 4) integrity at mucosal surfaces [70,71]. Therefore, worm therapy shows promise for broader application against allergies and autoimmune diseases in the future.

### 4.2 Nematodes for management of pest species

Several nematode species are already used as biocontrol agents to kill plant pests and thereby protect plant yield. Use of nematodes as alternative biocontrol agents to chemical pesticides can improve human and environmental health. The currently applied biocontrol species are entomopathogenic, meaning they infect their hosts and lyse them. Species that are used as biocontrol agents mainly belong to the genera *Steinernema* and *Heterorhabditis* as insect entomopathogens, while *Phasmarhabditis* is an entomopathogen of snails. Knowledge on entomopathogenic nematodes is being compiled in many studies and books [[72], [73], [74], [75]], all suggesting a high potential of nematodes to protect plants from pests and thereby reduce the application of artificial pesticides. Beyond the current application, entomopathogenic nematodes might also increase plant yield in presence of other pathogens such as shown for *Steinernema feltiae* by reducing root-feeding nematode infection [76]. Often the successful establishment of nematode biocontrol agents is limited or that the agents present in soils do not become sufficiently abundant or efficient. Different solutions are explored, like spiking with suitable prey species [77] or changing soil physicochemical properties [78], which likely will increase the efficacy of entomopathogenic nematodes in field settings. Finally, there is immense opportunity to expand the current use of nematodes as biocontrol agents by increasing the species diversity used and against different other pests.

### 4.3 Nematodes as bioindicators in natural soil and drinking water

Nematodes can be applied to monitor (harmful) environmental changes from an early stage. Nematode species differ in a wide variety of traits such as feeding, size and growth rate, that together result in differences in response to disturbance. The ease of extracting entire nematode communities from diverse environments (e.g. from different soil types and marine environments) allows reliable information about nematode community composition including specific abundance [79]. In addition, nematode identification with well-established morphological tools or sequence-based information along with deep-rooted expertise on species-specific traits continues to make nematodes unique as bioindicators [80]. The immense diversity of thousands of described species in addition to the advanced knowledge on nematodes has been used to create nematode community indices that predict factors including soil health, land use intensity and other management types [[81], [82], [83]]. Even emerging issues to environmental health such as microplastics and warming climate can be monitored with nematodes [84,85]. Together, the well-established indices make nematode communities an attractive target to evaluate quality of soil and water ecosystems.

Nematodes are also used as models in ecotoxicological assessments to determine the presence of pollution and human-adverse compounds such as heavy metals or organic compounds in drinking water [86,87]. Ecotoxicological assays are gaining importance as they are one of the first to detect negative effects of so-called ‘emerging’ compounds such as chemicals used in industry, residues of (new) medicines and pesticides. Emerging compounds are used (and discharged) before monitoring programs and norms are established. Different organism groups are used in ecotoxicological assays (e.g. springtails, earthworms, and water fleas), but nematodes in many ways surpass those in their use as models due to their ease of maintenance, high reproductive rates, limited resource requirements and well-established molecular tools. Classically, ecotoxicological tests assess the water quality based on reproduction, movement, or mortality of nematodes [86]. Yet, novel assays increasingly focus on more subtle effects such as feeding behaviour and gene expression patterns [86,88,89]. For example, toxic compounds in wastewater were discovered by highly sensitive genome-wide gene expression changes in the model nematode *C. elegans* [89,90]. Wastewater treatment essentially reversed all gene expression patterns back to regular [90], indicating that these combined molecular and fitness assays provide an attractive approach for monitoring toxic compounds in our environment.

## 5 Instrumental value of nematodes in science and education

Nematodes are at the basis of existing and future applications related to human health. Here, we describe why nematodes, as model organisms, have played a key role in facilitating discoveries related to human health and likely will do so in the future. In addition, we illustrate how the same qualities that make this group of organisms so easy to work with also offer exciting opportunities in educational settings.

### 5.1 Nematodes as model organisms in biology

Biological model species are studied in exceptional detail with the goal of being able to extrapolate findings from these organisms to others. One prominent model species is *C. elegans* that has greatly supported our understanding of metazoan (including human) molecular biology and health. There are many characteristics that make *C. elegans* a successful model: a rapid generation time (3 days) and the ability to propagate clonally through self-fertilization (resulting in about 300 offspring per parents) as well as being able to sexually reproduce. Vast gene-function screenings have been executed in this species, owing to its tractable genetics and the ability to be cryopreserved and revived even after a decade of being frozen. Formerly this was done by screening randomly induced mutations [91], but after 1998 targeted RNAi screenings were introduced [[92], [93], [94]]; and more recently by targeted gene knock-out using CRISPR/Cas9 [87,88].

The constantly expanding toolbox of genetic and molecular resources helped making *C. elegans* a universal model organism for human health [12]. The discovery of programmed cell death in this nematode backed our current genetic and molecular understanding of cancer [95]. Next to cancer research, *C. elegans* is used to understand a plethora of other health-related processes including cellular development, ageing, infectious disease, toxicology and the role of genetic individuality on disease development [12,83]. For example, the ability to create fluorescently tagged proteins with green fluorescent protein (GFP) [96] aided the transformation of *C. elegans* with a fluorescently-tagged human gene encoding alpha-synuclein, that is accumulating in the brain of people with Parkinson's disease [97]. Moreover, the availability of a highly complete and accurate genome sequence [98] enabled coupling various genes linked to behavioral ethanol responses [99] that are informative to our understanding of alcohol use disorders in humans [100].

Besides having a direct link to human health, *C. elegans* also serves as the first platform for establishing molecular techniques such as RNAi and CRISPR-Cas that can then be expanded towards non-model nematodes such as animal- and plant-parasitic nematodes [101,102]. The precisely annotated genome of *C. elegans* has often served as a reference for sequencing parasite species [103]. Moreover, *C. elegans* can be used to study functioning of genes with homologs in parasitic species. Established pathways in *C. elegans* can ease finding parallel pathways in other species, for example aiding the discovery of antiviral responses in animal-parasitic nematodes [104]. Finally, by using *C. elegans* as a model for other nematode species molecular and cellular mechanisms behind anthelmintic drug resistance were discovered [105].

### 5.2 Nematodes as teachers: applications in education

In education, nematodes can be used to teach a broad spectrum of biological concepts in life science domains ranging from cell biology and developmental biology [106,107] to fields such as behavioral biology [108] and ecology [109]. Exactly the characteristics that make nematodes attractive biological models ensure that nematodes are suitable for teaching various biological concepts in an educational setting. Considering that both time and budget are often limited, working with a model organism such as *C. elegans* can offer students the opportunity to work with living animals [106]. Moreover, students do not require special training or a license to perform experiments involving nematodes, in contrast to working with live vertebrate animals. Another characteristic that contributes to their suitability for educational activities is that nematodes are easy to work with and to observe for students at diverse educational levels [[109], [110], [111]]. Interestingly, nematode data collected by students can also be of real scientific value in citizen-science projects. This is illustrated by the CaeNDR database that contains hundreds of wild *Caenorhabditis* isolates, including *C. elegans* strains collected by non-scientists, and is used to understand natural genetic variation that is difficult to study in other organisms [112,113]. Combined, the qualities of nematodes allow students of different levels to work on inquiry-based science assignments that can include the entire experimental cycle, from design to interpretation [108,114].

## 6 Conclusions

Nematodes determine human health from highly diverse aspects. In this review, many positive effects of nematodes are discussed, but also negative influences are still common. To remain positive and improve negative nematode-human heath relationships in a changing world, novel approaches will be essential. Future studies could make use of this advanced understanding of molecular mechanisms behind parasitic nematode infections. Immunomodulatory properties of human and animal parasitic nematodes can form the basis of new therapies e.g. based on single molecules derived from nematodes (such as metabolites, glycoproteins or glycolipids). Understanding of molecular nematode-plant interactions can assist plant breeders to create environment-friendly approaches beyond single-resistance genes to safeguard food production. In addition, most nematode species and community networks are largely unexplored and offer many (perhaps unexpected) potential health benefits. A holistic approach could establish how nematode communities together provide health benefits to plants and targeted studies can unravel key species. Furthermore, genetic and molecular studies in *C. elegans* made this nematode one of the best examined organisms worldwide and comparisons between *C. elegans* and it's parasitic and free-living relatives can speed up research. Together, integrating different aspects of nematology, especially in education, will contribute to improved human health for the next generations.

## Funding

MGS was supported by 10.13039/501100003246NWO domain Applied and Engineering Sciences VENI grant (17282). JLL-T was supported by 10.13039/501100003246NWO domain Applied and Engineering Sciences VIDI grant (18389) and an Open Technology Programme grant (19977).

## CRediT authorship contribution statement

**Lisa van Sluijs:** Writing – review & editing, Writing – original draft, Project administration, Conceptualization. **Stefan Geisen:** Writing – original draft, Project administration, Conceptualization. **Jose L. Lozano-Torres:** Writing – original draft, Conceptualization. **Mark G. Sterken:** Writing – original draft, Visualization, Conceptualization. **Mariëtte T.W. Vervoort:** Writing – original draft, Conceptualization. **Ruud H.P. Wilbers:** Writing – review & editing, Writing – original draft, Project administration, Conceptualization.

## Declaration of competing interest

The authors declare that they have no known competing financial interests or personal relationships that could have appeared to influence the work reported in this paper.

## Data Availability

No data was used for the research described in the article.
